# Repositioning of Verrucosidin, a Purported Inhibitor of Chaperone Protein GRP78, as an Inhibitor of Mitochondrial Electron Transport Chain Complex I

**DOI:** 10.1371/journal.pone.0065695

**Published:** 2013-06-06

**Authors:** Simmy Thomas, Natasha Sharma, Reyna Gonzalez, Peng-Wen Pao, Florence M. Hofman, Thomas C. Chen, Stan G. Louie, Michael C. Pirrung, Axel H. Schönthal

**Affiliations:** 1 Department of Molecular Microbiology and Immunology, University of Southern California, Los Angeles, California, United States of America; 2 Department of Pharmaceutical Sciences, University of Southern California, Los Angeles, California, United States of America; 3 Department of Pathology, University of Southern California, Los Angeles, California, United States of America; 4 Department of Neurosurgery, University of Southern California, Los Angeles, California, United States of America; 5 Department of Clinical Pharmacy and Pharmaceutical Economics and Policy, University of Southern California, Los Angeles, California, United States of America; 6 Department of Chemistry, University of California Riverside, Riverside, California, United States of America; University of Hong Kong, Hong Kong

## Abstract

Verrucosidin (VCD) belongs to a group of fungal metabolites that were identified in screening programs to detect molecules that preferentially kill cancer cells under glucose-deprived conditions. Its mode of action was proposed to involve inhibition of increased GRP78 (glucose regulated protein 78) expression during hypoglycemia. Because GRP78 plays an important role in tumorigenesis, inhibitors such as VCD might harbor cancer therapeutic potential. We therefore sought to characterize VCD’s anticancer activity in vitro. Triple-negative breast cancer cell lines MDA-MB-231 and MDA-MB-468 were treated with VCD under different conditions known to trigger increased expression of GRP78, and a variety of cellular processes were analyzed. We show that VCD was highly cytotoxic only under hypoglycemic conditions, but not in the presence of normal glucose levels, and VCD blocked GRP78 expression only when glycolysis was impaired (due to hypoglycemia or the presence of the glycolysis inhibitor 2-deoxyglucose), but not when GRP78 was induced by other means (hypoxia, thapsigargin, tunicamycin). However, VCD’s strictly hypoglycemia-specific toxicity was not due to the inhibition of GRP78. Rather, VCD blocked mitochondrial energy production via inhibition of complex I of the electron transport chain. As a result, cellular ATP levels were quickly depleted under hypoglycemic conditions, and common cellular functions, including general protein synthesis, deteriorated and resulted in cell death. Altogether, our study identifies mitochondria as the primary target of VCD. The possibility that other purported GRP78 inhibitors (arctigenin, biguanides, deoxyverrucosidin, efrapeptin, JBIR, piericidin, prunustatin, pyrvinium, rottlerin, valinomycin, versipelostatin) might act in a similar GRP78-independent fashion will be discussed.

## Introduction

Verrucosidin (VCD) is a pyrone-type polyketide that is produced by several species of the genus Penicillium [1], [2]. It belongs to a group of tremorgenic mycotoxins that are known to act on the central nervous system, thereby causing tremors and convulsions with an intensity ranging from fully reversible without notable lesions to entirely fatal [3], [4]. Molds producing such tremorgens pose a recognized health risk to humans and animals when these microorganisms are present in spoiled food or feed. For example, livestock grazing on moldy pastures may develop sporadic locomotor incoordination, where they may stagger, fall over, and suffer muscular spasms [5], [6]. However, the mode of action for many of these mycotoxins has not been elucidated in detail.

It was reported recently [7] that VCD was able to kill human HT29 colon carcinoma cells in culture under glucose-deprived conditions. As well, VCD blocked the increased expression of glucose regulated protein 78 (GRP78) [7], a protein that is strongly induced during hypoglycemic stress and serves to ensure continued cellular survival under these conditions [8]. GRP78 is a key component of the unfolded protein response (UPR), a cellular process that is triggered in response to a variety of stress conditions that interfere with proper protein folding and processing in the endoplasmic reticulum (ER) [9]. For example, ER stress and subsequent activation of the UPR can be caused not only by hypoglycemia, but also by hypoxia and intracellular redox imbalance, as well as by a number of natural compounds, such as the sequiterpene lactone thapsigargin (which disturbes the intracellular calcium balance [10]) and the nucleoside antibiotic tunicamycin (which interferes with glycoprotein synthesis [11]). A common feature of these ER stressors is the ensuing accumulation of unfolded and misfolded proteins, and the risk of proteotoxicity. As an adaptive response, the UPR activates a set of pathways that result in the transcriptional activation of several important proteins, including GRP78, aimed at restoring proper protein processing and overall cellular homeostasis [12], [13], [14].

The identification of VCD as a GRP downregulator was through a screening assay based on the activity of a GRP78 promoter-luciferase reporter plasmid, where VCD prevented increased luciferase expression in response to glucose depletion or blockage of glycolysis with the hexokinase inhibitor 2-deoxyglucose (2-DG). A number of other compounds have also revealed GRP78 transcription inhibitory activity in this screening assay, such as arctigenin [15], biguanides (metformin, phenformin, buformin) [16], deoxyverrucosidin [17], efrapeptin J [18], analogs of JBIR [19], piericidin A [20], prunustatin A [21], pyrvinium [22], rottlerin [23], valinomycin [24], versipelostatin [25], and therefore collectively are considered GRP78 downregulators.

The prospect of using these compounds as specific inhibitors of GRP78 has raised much excitement and extensive interest [26], [27], [28], [29], because GRP78 is known to play key roles in cancer cells to ensure their continued proliferation under adverse conditions, such as hypoglycemia, hypoxia, acidosis, or accumulation of misfolded proteins [30]. Elevated levels of GRP78 are frequently documented in tumor tissue, where this protein contributes to cellular survival and resistance against certain types of chemotherapy [31], [32]. In breast cancer in particular, increased levels of GRP78 have been correlated with treatment resistance and poor prognosis for affected patients [33], [34], [35]. Therefore, GRP78 downregulators may harbor cancer therapeutic potential, and this aspect is deemed to warrant vigorous investigation [36].

Triple negative breast cancer (TNBC) is a subtype of breast cancer that lacks estrogen receptor and progesterone receptor expression, and does not overexpress epidermal growth factor receptor 2 (Her2/Neu) [37]. Because it carries poor prognosis and is not amenable to current targeted therapies, TNBC constitutes a problem with great need and urgency for improved therapies [38]. Like some other cancer types, TNBC cells are able to adapt to hypoglycemia and indeed continue to thrive under such microenvironmental conditions, and elevated basal level expression of GRP78 is among the contributing pro-survival factors under these otherwise hostile growth conditions [39].

The observation [7] that VCD exerted hypoglycemia-specific toxicity in colon carcinoma cells, possibly via the inhibition of GRP78, prompted us to hypothesize that this compound potentially might exert therapeutic benefit in TNBC. We therefore performed in vitro studies with representative human TNBC cell lines (MDA-MB-231 and MDA-MB-468) and characterized their response to de-novo chemically synthesized VCD. In the process, we discovered a hitherto unrecognized mechanism by which VCD accomplishes its cytotoxic outcome, namely by selectively blocking complex I of the respiratory chain and thus preventing mitochondrial ATP production.

## Materials and Methods

### Pharmacological Agents

Thapsigargin, tunicamycin, 2-deoxyglucose, oligomycin, 2-thenoyltrifluoroacetone (TTFA), and rotenone were purchased from Sigma-Aldrich (St. Louis, MO). Verrucosidin was de-novo chemically synthesized using the route described [40].

### Cell Lines and Culture Conditions

MDA-MB-231 and MDA-MB-468 human triple-negative breast cancer cell lines were obtained from the American Tissue Culture Collection (ATCC; Manassas, VA). They were propagated in DMEM (provided by the Cell Culture Core Lab of the USC/Norris Comprehensive Cancer Center and prepared with raw materials from Cellgro/MediaTech, Manassas, VA) supplemented with 10% fetal bovine serum, 2 mmol/L glutamine, 100 U/mL penicillin, and 0.1 mg/mL streptomycin in a humidified incubator at 37°C and a 5% CO_2_ atmosphere.

For conventional culture conditions, the medium contained standard 4.5 g/L glucose (25 mM). For hypoglycemic conditions, various lower glucose concentrations from 0.0 to 2.5 mM (as indicated in figure legends) were used. Hypoxic culture conditions were generated by placing the cells in a GasPak EZ Gas Generating Pouch System (BD Biosciences, San Jose, CA). In this system, the amount of oxygen is reduced to 0.7% within 2.5 h, and decreased further to below 0.1% by 24 h.

### MTT Assay

Methylthiazoletetrazolium (MTT) assays were performed as described earlier [41]. Briefly, cells were seeded into 96-well plates at 1.0 to 5.0×10^3^ cells per well and exposed to drug treatment (or solvent alone) for 48 or 72 h. In individual experiments, each treatment condition was set up in quadruplicate, and each experiment was repeated several times independently.

### Colony Formation Assay

Two hundred cells were seeded into each well of a 6-well plate. After cells had fully attached to the surface of the culture plate, they were exposed to drug treatment (or solvent alone) for 48 h. Thereafter, the drug was removed, fresh growth medium was added, and the cells were kept in culture undisturbed for 12–14 days, during which time the surviving cells spawned a colony of descendants. Colonies were visualized by staining for 4 h with 1% methylene blue (in methanol), and then were analyzed and counted using Image J software.

### Immunoblots

Total cell lysates were analyzed by Western blot analysis as described earlier [42]. The primary antibodies were purchased from Cell Signaling Technology (Beverly, MA), Santa Cruz Biotechnology, Inc. (Santa Cruz, CA) or Epitomics (Burlingame, CA) and used according to the manufacturers’ recommendations. All immunoblots were repeated at least once to confirm the results.

### Determination of Cellular Translation Rate

Cells were grown in 6-cm dishes under standard (25 mM glucose) or hypoglycemic culture conditions in the presence or absence of VCD or rotenone for 6 h. Then the monolayers were rinsed twice with phosphate buffered saline (PBS), and the growth medium was replaced with methionine-free medium supplemented with ^35^S-labeled L-methionine to a final activity of 10 µCi per mL of culture medium. The concentration of glucose and the respective drug treatment was maintained during this labeling period. The cells were cultured in the presence of ^35^S-methionine for 45 min. Thereafter, cells were harvested in PBS and transferred to a microcentrifuge tube. After a 30-second spin at 10,000 rpm, the buffer was aspirated and discarded, and the cell pellet lysed in 100 µL RIPA buffer. Protein concentration of each sample was determined, and 40 µg of total cell lysate was analyzed by polyacrylamide gel electrophoresis. After the run, the gel was stained with Coomassie blue, dried, and exposed to x-ray film.

### Cellular Bioenergetics Measurements

Cellular oxygen consumption rate (OCR) and extracellular acidification rate (ECAR) were measured with a Seahorse XF Extracellular Flux Analyzer (Seahorse Bioscience Inc., North Billerica, MA) according to the manufacturer’s instructions. Briefly, thirty thousand cells were seeded per well into a special 96-well plate. After overnight attachment, the medium was replaced with fresh medium with or without glucose. Basal levels of OCR and ECAR were recorded for 24 minutes, followed by the addition of VCD and continuous measurements for another hour.

Intracellular ATP levels were measured by using the ATP Colorimetric Assay Kit from Biovision (Milpitas, CA) according to the manufacturer’s instructions. Briefly, 10^6^ cells per 10-cm dish were exposed to treatment for various time points. Thereafter, cells were collected and lysed in 100 µL ATP assay buffer. ATP content in each lysate was determined in a reaction mix that supported the phosphorylation of added glycerol as a substrate. The reaction mix was incubated in a 96-well plate at room temperature for 30 minutes. The OD was measured at 570 nm in a microplate reader, and ATP content was calculated by applying sample readings to a standard curve.

### Analysis of Electron Transport Chain Components

The activity of complex I (NADH dehydrogenase) was analyzed in intact mitochondria that were isolated from mouse liver by using differential centrifugation as described previously [43]. It was assayed by monitoring the change in absorbance of the artificial electron acceptor dichlorophenolindophenol (DCIP) in the presence of NADH as previously described [44], [45] with slight modifications as follows. Twenty-three microliter of 0.2 M glycylglycine hydrochloride (pH 8.5), 13 µL of 0.6 mM DCIP, 6 µL of 6 mM NADH (prepared in 0.2 M glycylglycine buffer), and 48 µL of water were added to each well of a 96-well plate. Then, 50 µL of the drug to be tested was added. The reaction was started by the addition of 50 µg of isolated mitochondria to each well. Complex I activity was monitored by recording the change in absorbance over time at 600 nm for DCIP against reagent blank.

The activity of complex II was assayed in intact mitochondria by following a previously described method [46]. It was monitored using 2-(p-iodophenyl)-3-(p-nitrophenyl)-5-phenyl tetrazolium (INT) as the electron acceptor, which forms formazan crystals on reduction. Briefly, 20 µL of 0.5 M potassium phosphate (pH 7.4), 20 µL of 1% (w/v in 100% ethanol) INT, 20 µL of 0.5 M sodium succinate, 20 µL of 0.25 M sucrose, and 60 µL of water were added to each well of a 96-well plate. Then, 50 µL of the drug to be tested was added. The reaction was started by the addition of 50 µg of isolated mitochondria. The assay mixture was incubated at 37°C for 15 min to allow the reaction to proceed. The reaction was terminated by the addition of 50 µL of 10% trichloroacetic acid to each well, and the absorbance was read at 490 nm against reagent blank.

The activity of complex V was determined using the MitoTox Complex V OXPHOS Activity Microplate Assay kit from Abcam (Cambridge, MA), and manufacturer’s instructions were followed. Briefly, 50 µL of solubilized bovine heart mitochondria were added to each well of a pre-coated 96-well plate. The plate was incubated at room temperature for 2 hours. Thereafter, each well was washed twice with 300 µL of wash buffer. After washing, 40 µL of phospholipids were added to each well and the plate was incubated for 45 min at room temperature. At the end of the incubation period, the drugs to be tested were diluted in complex V activity solution and 200 µL of the dilution was added in quadruplicate to the pre-coated 96 well plate. The activity of complex V was measured by monitoring the change in absorbance at 340 nm over a period of 1 hour at 30°C.

### Statistical Analysis

All parametric data were analyzed using the Student t-test to calculate the significance values; a probability value (*p*) <0.05 was considered statistically significant.

## Results

### VCD is Cytotoxic under Hypoglycemic Conditions

**Figure 1:**
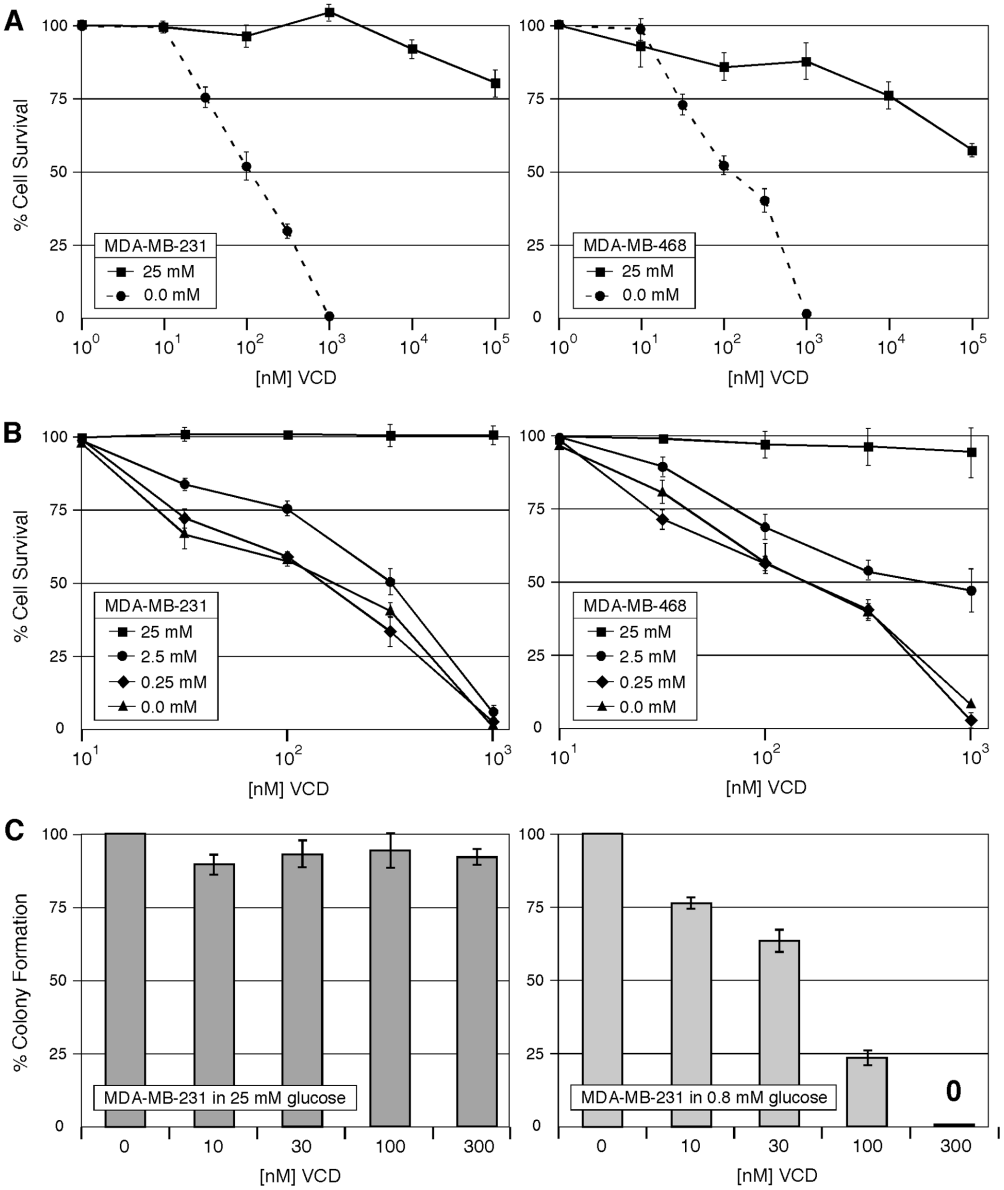
Cell growth inhibition by VCD. MDA-MB-231 and MBA-MD-468 cells were exposed to VCD treatment in cell culture. (A) Cells were treated with increasing concentrations of VCD in the absence (0.0 mM) or presence (25 mM) of glucose for 48 h and cell viability was determined by MTT assay. Removal of glucose by itself (in the absence of VCD) did not substantially interfere with cell viability during this 48-hour period (not shown). (B) Cells were exposed to VCD in the presence of various concentrations of glucose (as indicated) for 48 h and cell viability was determined by MTT assay. (C) Cells were exposed to increasing concentrations of VCD in the presence of 25 mM (left panel) or 0.8 mM (right panel) glucose for 48 h; thereafter, VCD was removed and culture conditions were continued in 25 mM glucose for all cells. Long-term cell survival was determined by counting the number of emerging colonies two weeks later. In all cases, survival of untreated control cells was set at 100%. All values are n≥3 (±s.d.).

Two triple-negative breast cancer cell lines, MDA-MB-231 and MDA-MB-468, were exposed to increasing concentrations of VCD in the presence or absence of glucose, and cell survival was investigated. As determined by MTT assay, VCD was highly cytotoxic in the absence of glucose, with an IC50 of about 120 nM in both cell lines (**Fig. 1A**). In stark contrast, VCD concentrations of up to 100,000 nM were only weakly effective and killed less than 50% of the cells under normal (25 mM glucose) culture conditions. Complete absence of glucose was not necessary for VCD to unfold its potent cytotoxicity, as this compound very effectively reduced cellular survival in the presence of 0.25 and 2.5 mM glucose as well (**Fig. 1B**).

We also determined long-term cellular survival by colony formation assay. Here, VCD proved even more cytotoxic under hypoglycemic (0.8 mM glucose) conditions than in the above short-term MTT assays; 300 nM completely blocked the formation of any colonies, and the IC50 was well below 100 nM (**Fig. 1C**). In comparison, in the presence of 25 mM glucose, VCD up to 300 nM had no inhibitory effect on colony formation. Altogether, these results demonstrate a huge differential in VCD’s cytotoxic potency that is dependent on hypoglycemic conditions.

### VCD Blocks GRP78 Induction during Hypoglycemia

**Figure 2:**
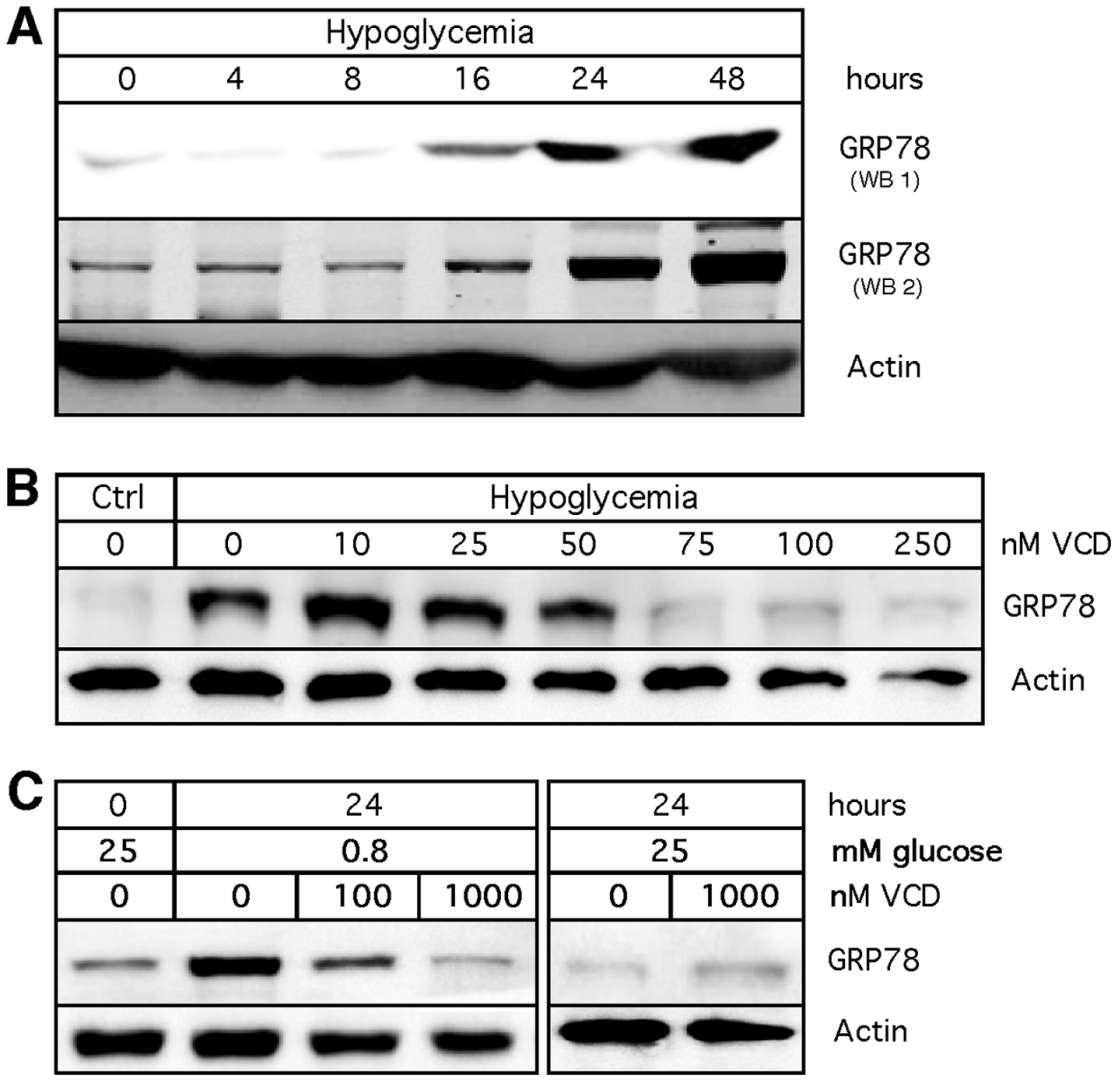
Inhibition of GRP78 induction by VCD. GRP78 protein expression was analyzed by Western blot analysis of MDA-MB-231 cells. (A) Cells were exposed to hypoglycemic (0.8 mM glucose) culture conditions for up to 48 h. Shown are two independent repeats (WB1, WB2) with different exposure times. (B) Cells were exposed to hypoglycemic culture conditions in the absence or presence of increasing concentrations of VCD for 24 h. Ctrl: cells were kept in normal (25 mM glucose) culture conditions. (C) Cells were treated with up to 1000 nM VCD under hypoglycemia or in the presence of 25 mM glucose for 24 h. In all cases, blots were re-probed with an antibody against actin (as a loading control).

GRP78 is a chaperone protein that supports cellular survival during hypoglycemic conditions. As expected, our hypoglycemic culture conditions caused strong elevation of GRP78 protein levels (**Fig. 2A**). When we exposed cells to hypoglycemia in the presence of increasing concentrations of VCD, we found that VCD concentrations as low as 75 nM potently blocked the elevation of GRP78 protein levels, and the amount of actin protein began to diminish under these conditions (**Fig. 2B**) (see Discussion section for an interpretation of the actin response). Under normal (25 mM glucose) conditions, VCD effects on GRP78 could not be observed: because there is no increase in GRP78 levels in the presence of normal amounts of glucose, it follows that the presence of VCD, even at a concentration of 1,000 nM, naturally had no target for blockage (**Fig. 2C**).

**Figure 3:**
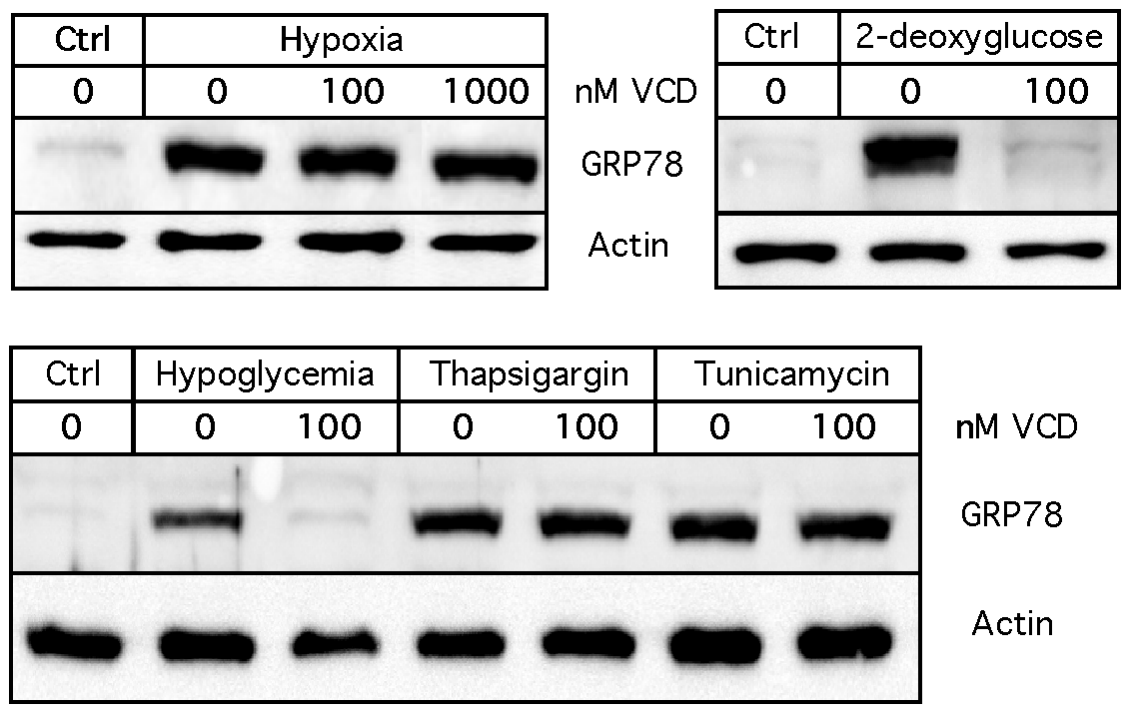
Differential inhibition of GRP78 induction by VCD. Cells were exposed to a variety of known inducers of the ER stress response in the absence or presence of VCD for 24 hours. 2-Deoxyglucose was used at 20 mM; thapsigargin and tunicamycin were used at 200 nM. GRP78 protein expression was analyzed by Western blot analysis. In all cases, blots were re-probed with an antibody against actin (as a loading control). Ctrl: untreated cells kept under normal culture conditions. Ctrl: cells were kept in normal (25 mM glucose) culture conditions without drug treatment.

As it is known that GRP78 protein expression is strongly increased not only in response to lower glucose levels, but more generally also by a great variety of conditions that cause endoplasmic reticulum (ER) stress, we next investigated additional triggers of ER stress. Cells were exposed to hypoxia, 2-deoxyglucose (2-DG, an inhibitor of hexokinase), thapsigargin (an inhibitor of SERCA, i.e., sarcoplasmic/endoplasmic reticulum calcium ATPase), and tunicamycin (an inhibitor of protein glycosylation) in the presence or absence of VCD. As shown in **Fig. 3**, VCD did not block GRP78 induction in response to hypoxia, thapsigargin, or tunicamycin; however, it did block GRP78 induction in response to 2-DG. Thus, altogether VCD blocked GRP78 induction under conditions that affected glycolysis, namely hypoglycemia and hexokinase inhibition by 2-DG, but it had no effect on GRP78 induction when other ER stressors were used.

### Hypoxia Displays Effects Similar to VCD

**Figure 4:**
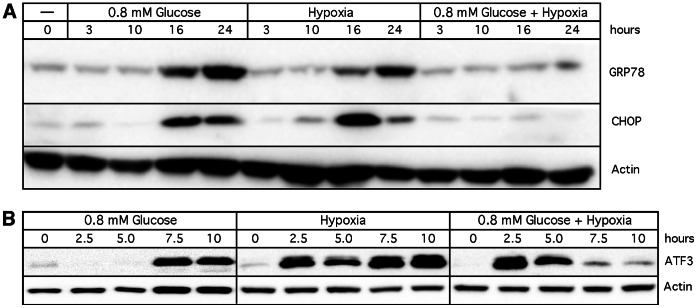
Combination effect of hypoglycemia and hypoxia. Cells were exposed to hypoglycemia or hypoxia, or both. At different time points, cells were harvested and Western blot analysis was performed. (A) GRP78 and CHOP expression levels over 24 hours. (B) ATF3 expression levels during 10 hours of treatment. Actin was used as a loading loading control.

Increased levels of GRP78 represent a reliable marker for ER stress, but other proteins participate in the ER stress response as well. Among them is CHOP (CCAAT/enhancer binding protein homologous protein), which is critical for the pro-apoptotic function of this cellular program [47]. As well, ATF3 (activating transcription factor 3) is a general indicator of cellular stress [48]. In order to investigate the mechanism underlying the differential effects of VCD during hypoglycemia (inhibition of GRP78) vs. hypoxia (no inhibition of GRP78), we wanted to add VCD to cells under dual treatment with hypoglycemia plus hypoxia, and then investigate the stress markers GRP78, CHOP, and ATF3. First, expression of these three markers was analyzed in the absence of VCD. To our surprise, and as shown in **Fig. 4A**, dual treatment with hypoglycemia plus hypoxia did not result in increased expression of GRP78 or CHOP, even though individual treatment with either condition triggered greatly elevated levels of these proteins. In the case of ATF3, we focused on earlier time points because stimulation of this general stress marker takes place more rapidly as compared to GRP78 or CHOP, and greatly elevated levels were present as early as 2.5 hours (hypoxia) and 7.5 hours (hypoglycemia) (**Fig. 4B**). The response of ATF3 to dual treatment, however, was intriguing: while there was an initial increase, it was followed by a strong decline, as if the cells ran out of sufficient energy to mount a further response (**Fig. 4B**).

The lack of increased GRP78 (and CHOP and ATF3) levels under dual (hypoglycemia plus hypoxia) treatment condition precluded the intended addition of VCD to this condition. However, this outcome was remarkable because it suggested that hypoxia might mimic the effects of VCD, i.e., hypoxia blocked the strong increase in GRP78 expression that normally takes place under hypoglycemic conditions. Because hypoglycemia suppresses energy production from glycolysis, and hypoxia impedes energy production from mitochondria, the above result hinted at the possibility that VCD, rather than acting as a specific inhibitor of GRP78, might repress energy production from mitochondria.

### VCD Inhibits General Cellular Translation

**Figure 5:**
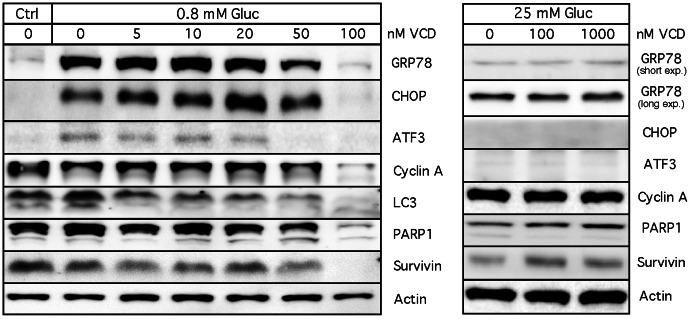
Various protein levels affected by VCD. Cells were cultured in the presence of 0.8 or 25 mM glucose, in the absence or presence of increasing concentrations of VCD for 24 h. Expression levels of various proteins were analyzed by Western blot analysis. The right panel contains two different exposures of the GRP78 blot; the short exposure is equivalent to the exposure shown in the left panel, whereas the longer exposure is included to prominently display the low basal levels of this protein (revealing the absence of an inhibitory effect of VCD). Ctrl: cells were kept in normal (25 mM glucose) culture conditions.

Based on the assumption that VCD might restrict mitochondrial energy production, its inhibitory effects on GRP78 induction under hypoglycemic conditions should not be specific, but should also become evident with other inducible proteins. We therefore analyzed the expression levels of CHOP and ATF3, which also represent hypoglycemia-responsive and ER stress-inducible proteins. As shown in **Fig. 5**, hypoglycemia caused increased expression of CHOP and ATF3, and this increase was effectively blocked by 100 nM VCD, similar to what was observed in the case of GRP78. Basal level expression of a number of other, non-hypoglycemia-responsive proteins was down-regulated by VCD as well; among those were cyclin A (a cell cycle regulator), LC3 (a component of autophagy), PARP1 (involved in DNA repair and cell survival), and survivin (an anti-apoptotic protein). There was also a small effect on actin (as noted above in **Fig. 2**; see Discussion section for an interpretation of the actin response). In contrast, under normal (25 mM glucose) culture conditions, neither cyclin A nor PARP1 nor survivin basal levels were downregulated by VCD, even at concentrations of up to 1,000 nM. Inhibition of induction of GRP78, CHOP, and ATF3 cannot be investigated in the presence of 25 mM glucose, because their expression levels are not stimulated in the presence of plentiful amounts of glucose. Instead, we investigated the effects of VCD on basal level (unstimulated) expression of GRP78 by long-term exposure of the Western blot (to prominently expose the low levels of GRP78). This revealed that VCD at up to 1,000 nM exerted no inhibitory effect on GRP78 in the presence of glucose (**Fig. 5**, right panel). Altogether, these results demonstrate that VCD is able to down-regulate the expression of a number of genes, not only GRP78, under conditions of low glucose supply; however, in the presence of plentiful glucose, none of these targets, inclusive of GRP78, are affected.

**Figure 6:**
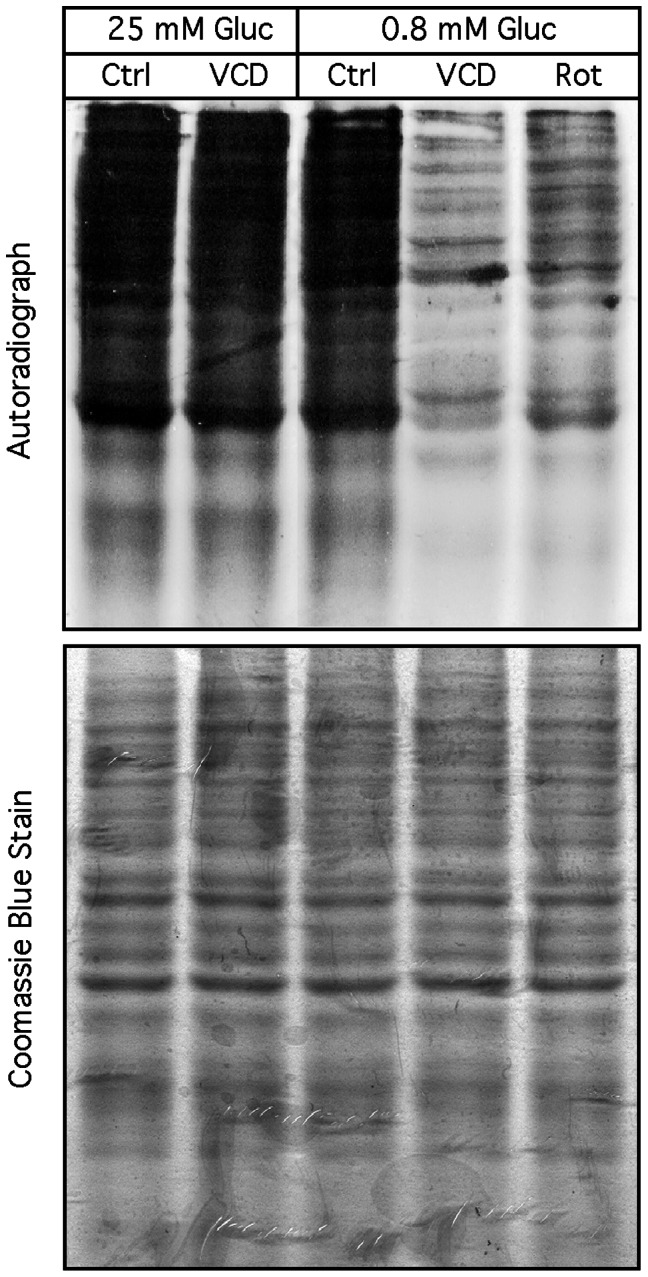
Inhibition of global cellular translation by VCD. Cells were exposed to 100 nM VCD under hypoglycemic (0.8 mM glucose) or normal (25 mM glucose) culture conditions. In parallel, hypoglycemic cells were treated with 100 nM rotenone. After 6 h, the rate of ongoing protein synthesis was determined. The top panel (autoradiograph) shows greatly reduced levels of incorporated ^35^S-labeled amino acids (indicating severely diminished translational activity) when VCD or rotenone are present under hypoglycemic conditions. The bottom panel shows Coomassie blue staining of the same gel to confirm equal overall amounts of proteins present. Ctrl: cells were kept in normal (25 mM glucose) culture conditions without drug treatment.

Because the above results suggested an inhibitory effect of VCD on a great number of proteins, not only GRP78, we analyzed total cellular protein synthesis by measuring overall translational activity. Cells were treated with VCD, or the respiratory chain blocker rotenone, in the presence of 25 mM glucose or under hypoglycemic culture conditions for 6 h; thereafter, translational activity was determined via the incorporation of ^35^S-methionine into newly synthesized proteins. As shown in **Fig. 6**, VCD did not noticeably affect incorporation of ^35^S-methionine in the presence of normal glucose levels; as well, hypoglycemia by itself did not reduce ^35^S-methionine incorporation, which was consistent with our observation that these tumor cells are fairly resistant to glucose starvation (not shown). However, VCD did cause a pronounced (about 80%) decrease in ^35^S-methionine incorporation under hypoglycemic conditions, and this inhibitory effect was very similar in hypoglycemic cells treated with rotenone. Thus, VCD sharply reduced overall protein synthesis under hypoglycemic conditions, revealing that VCD inhibited the expression of a very large number of proteins, not only GRP78, exclusively under hypoglycemic conditions. Intriguingly, the mitochondial inhibitor rotenone appeared to mimic the effects of VCD, further indicating the possibility that VCD might impinge on cellular energy production.

### VCD Inhibits Mitochondrial Energy Production

**Figure 7:**
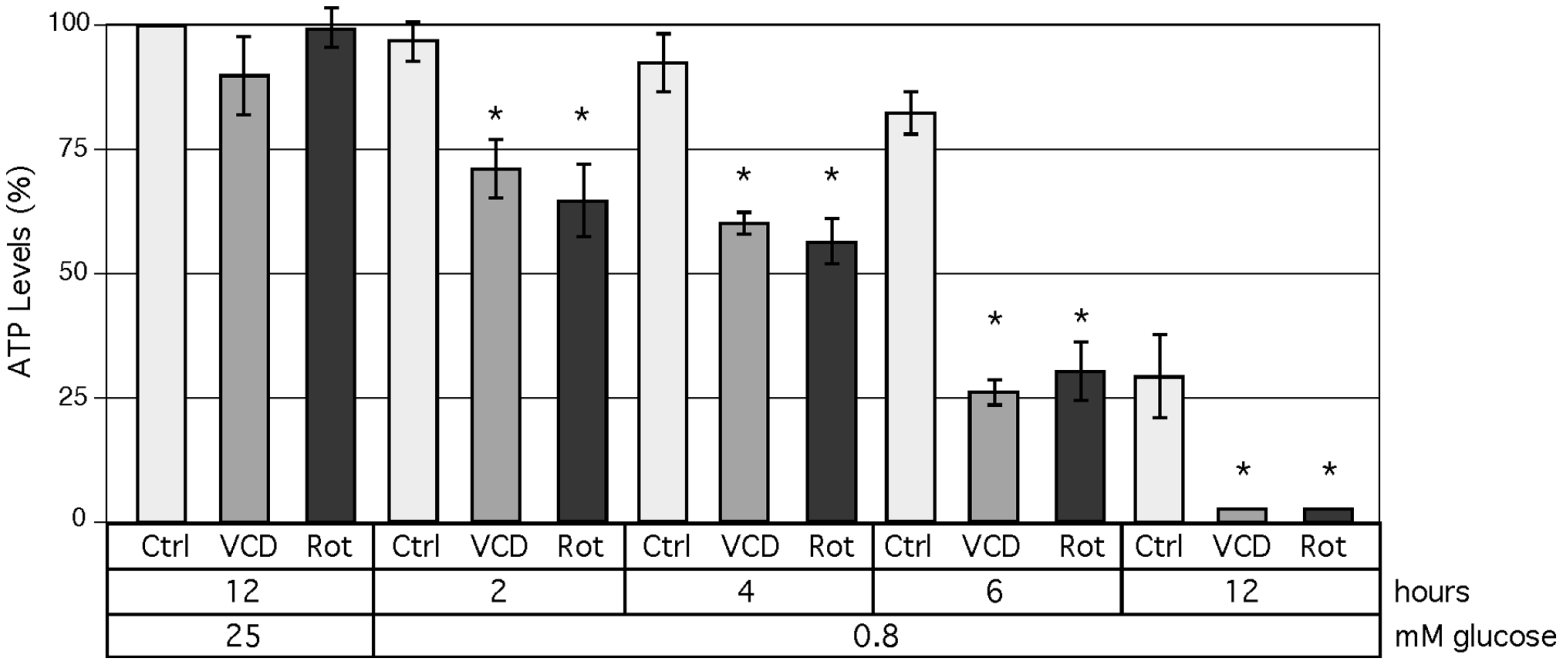
Inhibition of ATP synthesis by VCD. Intracellular levels of ATP were determined after incubation of cells under various culture conditions. Cells were exposed to 100 nM VCD or 100 nM rotenone in the presence of 0.8 or 25 mM glucose for up to 12 h. Ctrl: control for the respective time point in the absence of drug treatment. Asterisks: *p*<0.05 (treated compared to untreated cells at the same time point).

To determine VCD’s postulated effects on energy production more directly, we measured intracellular ATP levels after treatment of cells with VCD or rotenone in the presence or absence of glucose. As shown in **Fig. 7**, neither VCD nor rotenone displayed pronounced effects on ATP levels when cells were kept in the presence of normal glucose levels. However, in the absence of glucose both compounds drastically lowered intracellular ATP levels. Hypoglycemia by itself also lowered ATP levels over time (to 30% at 12 hours), but this decline was substantially slower and much less severe than in the presence of VCD or rotenone (<5%). As shown by earlier studies (for examples, see [49], [50]), ATP decline to about 20% can be tolerated by cells for extended periods (weeks), especially when growth factors/serum are present; however, if levels drop substantially lower, cellular adaptation processes (inclusive of autophagy [51]) are unable to compensate and cells will die (e.g., [52], [53]). Therefore, the observed massive depletion of ATP levels by VCD under hypoglycemic condition is consistent with general shutdown of cellular functions (inclusive of global gene expression, **Fig. 5**) and cell death due to energy exhaustion.

**Figure 8:**
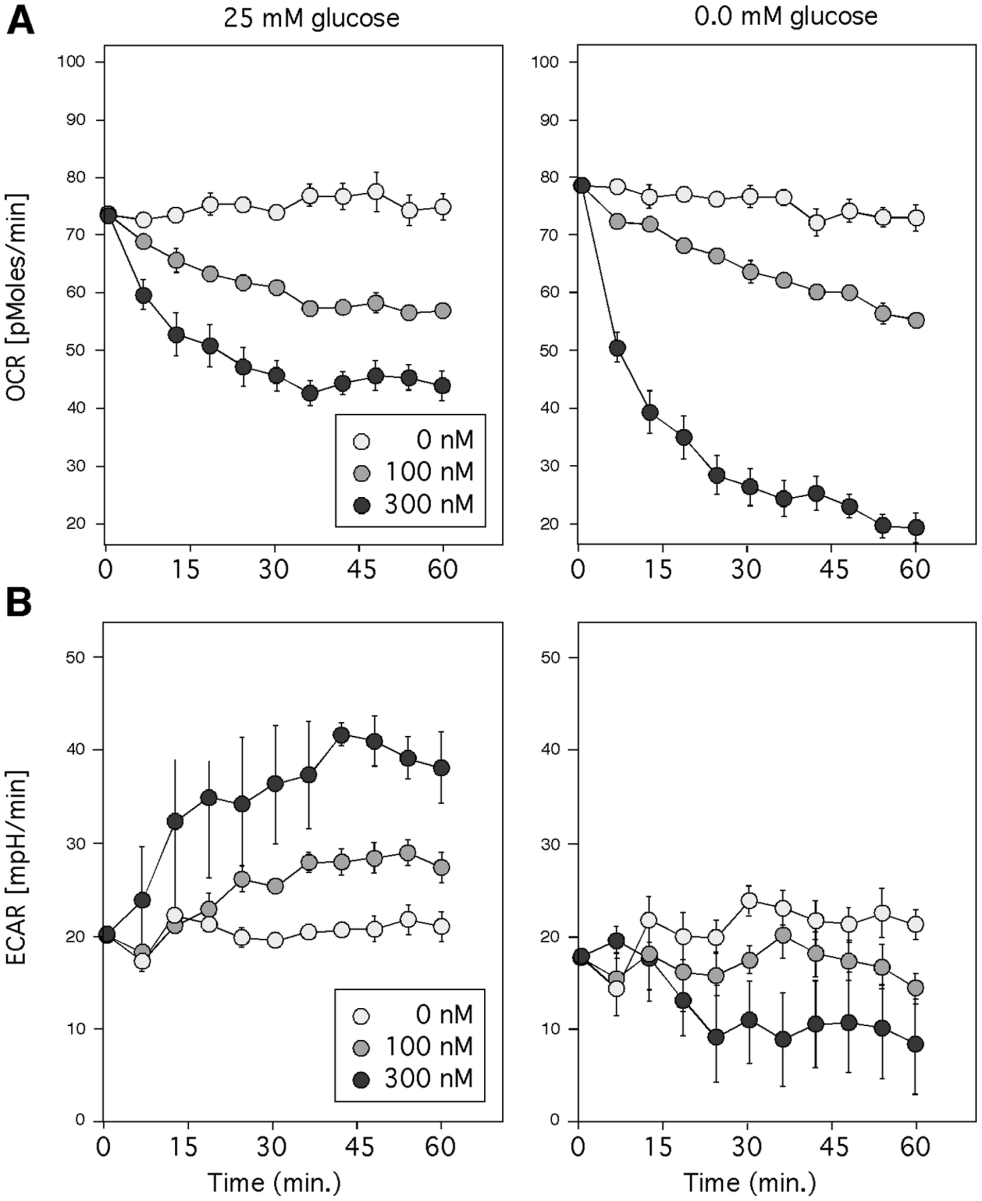
Metabolic effects of VCD. Cells were exposed to increasing concentrations of VCD in the absence (0.0 mM) or presence (25 mM) glucose. (A) Mitochondrial respiration was analyzed by measuring the oxygen consumption rate (OCR; pMoles/min). (B) Glycolytic flux was quantitated by measuring extracellular acidification rate (ECAR; mpH/min).

The bioenergetic effects of VCD were investigated further with the use of the Seahorse analyzer. This piece of equipment is able to quantify mitochondrial respiration by measuring the oxygen consumption rate (OCR); as well, it can quantify glycolytic flux by measuring the extracellular acidification rate (ECAR). Cells were exposed to 100 and 300 nM VCD in the presence and absence of glucose, and OCR and ECAR were measured at 6-minute intervals for up to one hour. The results are summarized in **Fig. 8** as follows.

VCD dose-dependently decreased OCR in the presence and in the absence of glucose. While inhibition by 100 nM was fairly similar under both conditions, 300 nM VCD resulted in greater OCR inhibition in the absence of glucose. It should be noted, however, that this latter readout might overestimate VCD’s inhibitory effect, because in the absence of glucose 300 nM VCD is much more toxic, which might artificially decrease OCR even further. Nonetheless, combined these data reveal substantially impaired mitochondrial respiration in the presence of VCD. On the other hand, measuring ECAR revealed an up to 50% increase when VCD was added to glucose-rich medium, whereas addition of VCD to medium lacking glucose did not result in any ECAR increase. (In the latter case, 300 nM VCD caused diminished ECAR, which we suspect might be related to increased toxicity at this high VCD concentration.) In any case, the ECAR data indicate that glycolytic flux is greatly increased when mitochondrial respiration is impaired by VCD, and, as would be expected, this outcome only takes place in the presence of glucose.

### VCD Inhibits Complex I of the Respiratory Chain

**Figure 9:**
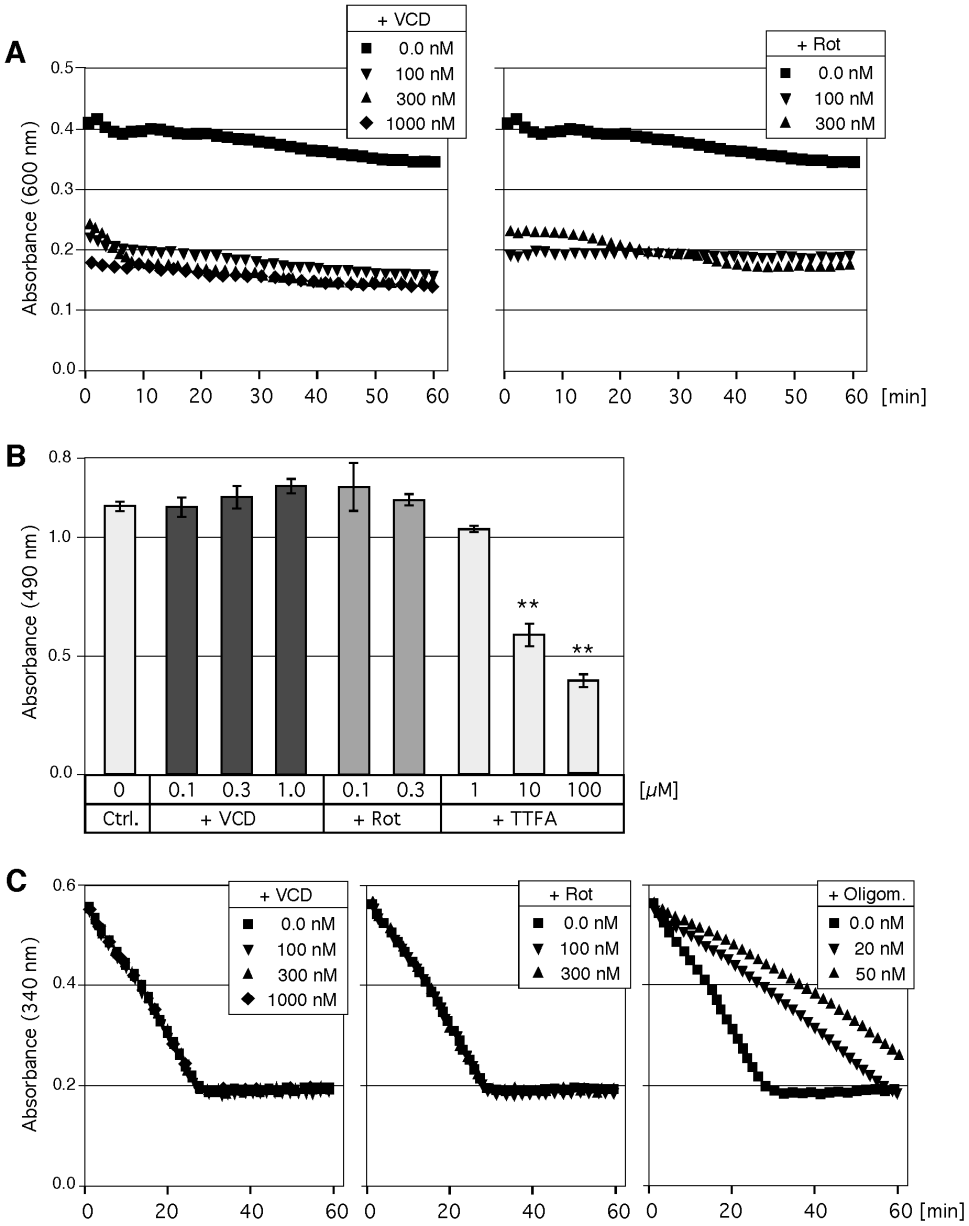
Mitochondrial target of VCD. The activities of specific complexes of the electron transport chain were analyzed in intact mitochondria in the presence and absence of drug treatment. (A) Complex I activity was recorded as absorbance (600 nm) of the electron acceptor DCIP over time. Note that inhibition of activity by nanomolar concentrations of VCD or rotenone (Rot) resulted in prominently lower absorbance. (B) Complex II activity was recorded as absorbance (490 nm) of formazan formed after reduction of electron acceptor INT. Note that neither VCD nor rotenone affected complex II activity (none of the minor variations was statistically significant, i.e., the *p*-value was greater than 0.05 in all two-sided comparisons). The known complex II inhibitor TTFA served as the positive control and significantly (*p*<0.01; asterisks) inhibited complex II activity. Ctrl: control, i.e., value in the absence of drug treatment. (C) Mitochondrial ATP synthase (complex V) activity was recorded as a decrease in absorbance (340 nm) over time. Note that ATP synthase inhibitor oligomycin inhibits complex V activity (used as a positive control; right panel), but neither VCD nor rotenone show inhibitory effect. All assays A-C were repeated at least once and yielded essentially the same outcomes.

Because of VCD’s impairment of mitochondrial respiration, we next set out to measure the activity of specific complexes within the electron transport chain. In particular, we analyzed the effects of VCD on complex I, complex II, and complex V (ATP synthase). As summarized in **Fig. 9**, VCD potently blocked complex I, but not complex II or complex V. Inhibition of complex I appeared to be maximal at 100 nM, and further increased concentrations of VCD (300 and 1,000 nM) exerted only marginally stronger effects. Intriguingly, blockage of complex I activity by VCD was as effective as blockage achieved by the classic complex I inhibitor rotenone, which was used as a positive control (**Fig. 9A**).

**Fig. 9B** demonstrates that VCD did not impinge on complex II activity, even at concentrations up to 1,000 nM. Similarly, rotenone did not affect this complex either, whereas the established complex II inhibitor TTFA, which was used as a positive control, led to significant inhibition of this complex. As well, neither VCD nor rotenone affected the activity of complex V (**Fig. 9C**). In contrast, oligomycin, a known inhibitor of complex V that was used as a positive control, significantly blocked complex V activity (**Fig. 9C**). Altogether, these results identify VCD as a potent inhibitor of complex I of the electron transport chain, with similar potency as rotenone.

### Rotenone Mimics VCD Effects

**Figure 10:**
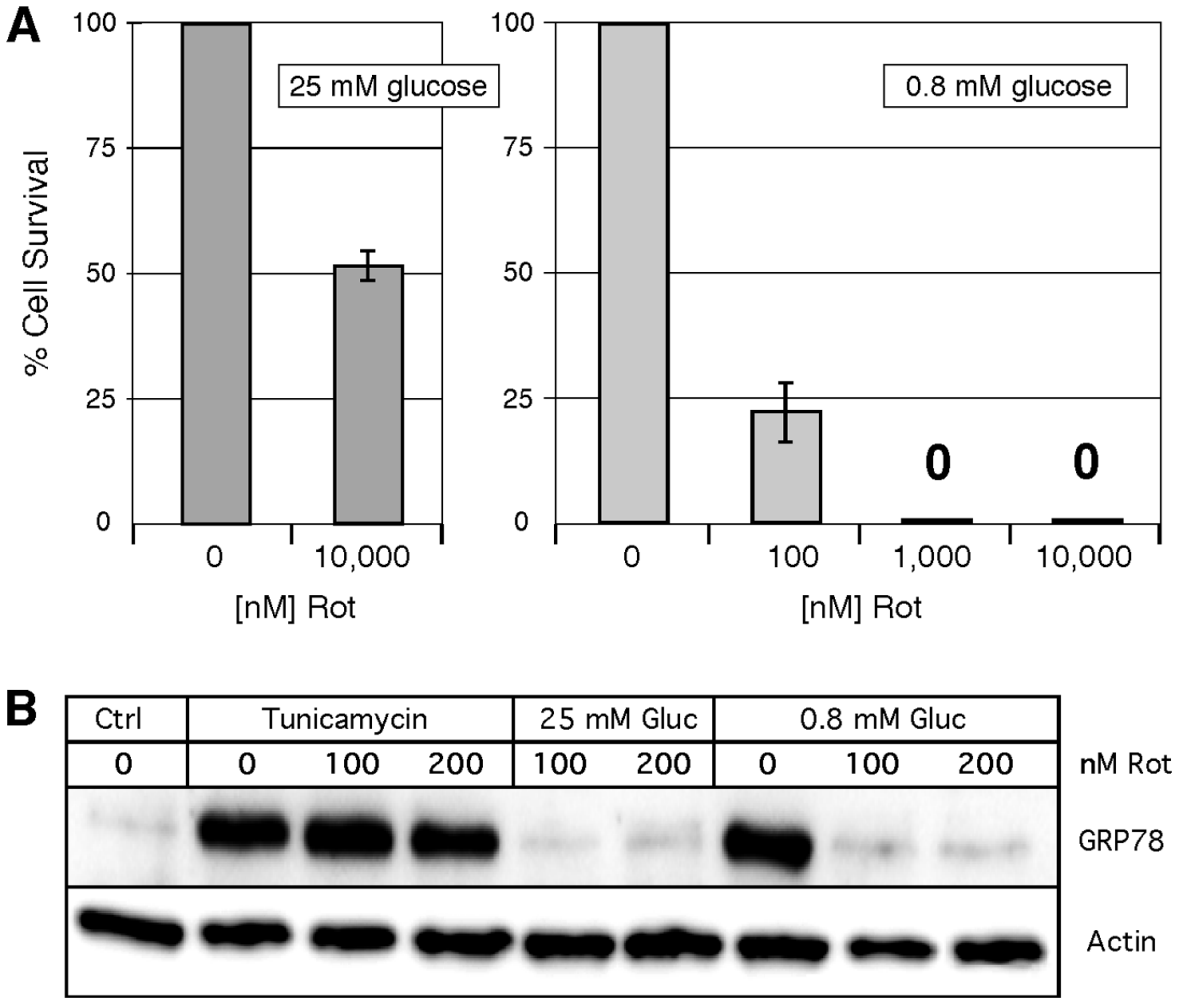
Mimicry of VCD effects by rotenone. Cells were exposed to increasing concentrations of rotenone (Rot) under hypoglycemic (0.8 mM glucose) conditions or in the presence of 25 mM glucose. (A) After 48 h, cell viability was determined by MTT assay. Survival of cells in the absence of Rot was set to 100%. (B) Cells were treated with 200 nM tunicamycin (in the presence of 25 mM glucose), were exposed to hypoglycemia (0.8 mM glucose), or were kept under normal (25 mM glucose) culture conditions, and in all cases 100 or 200 nM Rot was added. After 24 h, cell lysates were analyzed by Western blot for GRP78 expression. Actin was used as a loading control. Ctrl: cells were kept in normal (25 mM glucose) culture conditions without any drug treatment.

If the molecular and cellular effects of VCD were primarily caused by its blockage of complex I activity, one would hypothesize that the established complex I inhibitor rotenone should be able to trigger the same outcomes. This possibility already was suggested by our observation that rotenone inhibited ATP synthesis and general protein translation under hypoglycemic conditions very similarly to VCD (**Fig. 6** and **Fig. 7**). We therefore investigated whether rotenone would be able to mimic other VCD effects as well. This was indeed the case. As shown in **Fig. 10A**, rotenone also exerted hugely differential ability to kill cells depending on the glucose concentration; while 100 nM was highly cytotoxic under low-glucose conditions and reduced cell viability by over 75%, even 100-times higher concentrations were significantly less toxic in the presence of normal glucose levels.

Rotenone faithfully mimicked VCD’s effects on GRP78 expression as well. It effectively blocked GRP78 induction in response to hypoglycemia, but had no effect on GRP78 induction in response to tunicamycin (**Fig. 10B**). Taken together, the effects of the electron transport chain blocker rotenone and the effects of the purported GRP78 downregulator VCD were indistinguishable in our experiments, in essence qualifying rotenone as a GRP78 downregulator, or conversely VCD as a mitochondrial inhibitor.

**Figure 11:**
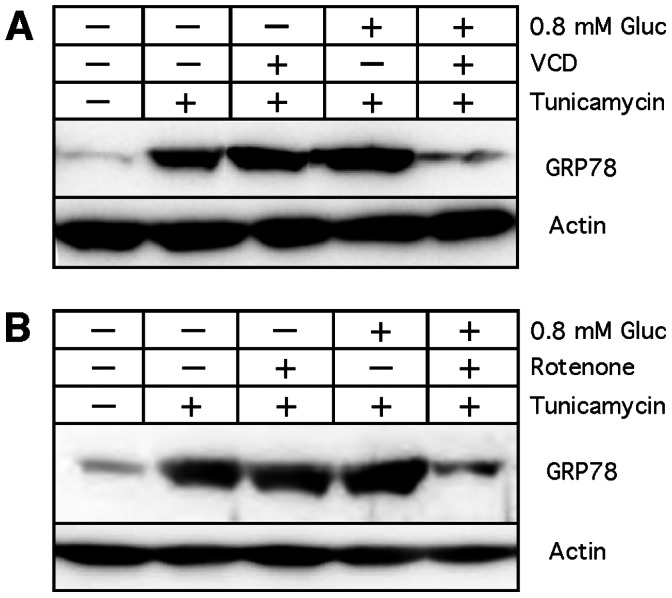
Inhibition of tunicamycin-induced GRP78 expression by VCD and rotenone. Cells were exposed to hypoglycemia (0.8 mM glucose) in the presence or absence of 100 nM VCD (A) or 100 nM rotenone (B) at time 0 hours. In all cases, tunicamycin was added 8 hours later for an additional 16 hours. Thereafter, cell lysates were prepared and analyzed by Western blot for GRP78 expression levels. Actin was used as a loading control.

The above results indicated that VCD (or rotenone) blocked GRP78 induction under hypglycemic conditions because combined blockage of the two cellular energy sources (glycolysis and mitochondria) precluded the availability of sufficient ATP (**Fig. 7**) for the synthesis of GRP78 (and many other proteins, **Fig. 6**). In comparison, neither VCD nor rotenone blocked GRP78 induction by tunicamycin (**Fig. 3**, **Fig. 10**), presumably because ongoing glycolysis in the presence of plentiful glucose provided sufficient energy for protein synthesis to take place. To furnish further evidence for this conjecture, we pre-treated cells with hypoglycemia+VCD (or hypoglycemia+rotenone) for 8 hours before the addition of tunicamycin. This pre-treatment, which strongly reduces cellular ATP levels (**Fig. 7**), would then be expected to prevent GRP78 induction by tunicamycin. As shown in **Fig. 11**, this was indeed the case. While pre-treatment of cells with VCD alone or hypoglycemia alone did not interfere with GRP78 induction by tunicamycin, the combination of hypoglycemia+VCD greatly minimized tunicamycin’s ability to stimulate GRP78 expression (**Fig. 11A**). As before, rotenone was able to faithfully mimic VCD’s effect (**Fig. 11B**).

## Discussion

VCD belongs to a group of natural compounds that have revealed strict hypoglycemia-dependent cytotoxicity and the ability to block the increased expression of GRP78 that occurs in response to hypoglycemia. Other members of this group are arctigenin [15], biguanides (metformin, phenformin, buformin) [16], deoxyverrucosidin [17], efrapeptin J [18], analogs of JBIR [19], piericidin A [20], prunustatin A [21], pyrvinium [22], rottlerin [23], valinomycin [24], and versipelostatin [25]. Because GRP78 plays an important role in tumorigenesis, such inhibitors might harbor cancer therapeutic potential. However, based on our present study with VCD as a representative of this group of GRP78 inhibitors, the presumed mechanism of antitumor action of at least some of these compounds should be reconsidered. The results from our experiments strongly suggest that (i) VCD kills tumor cells via inhibition of mitochondrial oxidative phosphorylation (OXPHOS), and (ii) VCD’s inhibitory effect on GRP78 represents an entirely non-specific consequence secondary to cytotoxic ATP depletion.

VCD treatment of hypoglycemic cells prevents cellular ATP synthesis (Figure 7). This combination of hypoglycemia ( = inefficient ATP synthesis from glycolysis) with VCD ( = blockage of oxidative phosphorylation) leaves no compensatory mechanism for ATP production, and insufficient amounts of ATP cause deterioration of critical cellular processes (such as general translation, Figure 6; and presumably transcription as well). In the absence of translation, there is no new synthesis of cellular proteins, which has two consequences: (a) there is no stimulation (i.e., no increased levels) of hypoglycemia-inducible proteins (GRP78, CHOP, ATF3, etc.) (Figure 5), and (b) there is a decline in the levels of highly abundant proteins (cyclin A, LC3, survivin, PARP1, etc.) over time (Figure 5); we also noted a decline in the amount of (the very long-lived) actin protein, which becomes more prominent at longer incubation times (not shown). Thus, VCD non-specifically blocks the expression of very many proteins, not just GRP78.

It is important to consider that VCD (and all other purported GRP78 downregulators of this group) was identified as an inhibitor of GRP78 *transcription* (i.e., via the inhibition of a hypoglycemia-stimulated GRP78 promoter-luciferase reporter plasmid), and that this inhibitory effect is specific for hypoglycemia and does not take place when GRP78 transcription is stimulated by other types of ER stress. For example, VCD (Figure 3) and other GRP78 downregulators (arctigenin, efrapeptin J, metformin, pyrvinium, versipelostatin) block GRP78 transcription during hypoglycemia (or 2-DG treatment), but not when GRP78 is stimulated by thapsigargin, tunicamycin, or A23187 [15], [16], [18], [22], [25]. This very peculiar differential can be rationally explained via ATP depletion, which occurs during hypoglycemia (Figure 7) but not during other types of ER stress that do not involve impaired glycolysis.

If hypoglycemia-specific cell killing by purported GRP78 downregulators indeed were mediated primarily via mitochondrial effects, as we claim, one would assume that other means of mitochondrial impairment would be able to mimic these effects. This is indeed the case. (i) We found that rotenone, a classic inhibitor of the electron transport chain (ETC), is hugely more toxic under hypoglycemic conditions than in the presence of high glucose levels, and blocks GRP78 induction under hypoglycemia, but not in response to tunicamycin–just like VCD (Figure 10). (ii) Hypoxia, which impedes mitochondrial activity, achieves this outcome as well (Figure 4). (iii) Mitochondrial DNA-deficient (rho-minus) cells do not tolerate hypoglycemia (or 2-DG) and do not mount an ER stress response under these conditions, but do mount a normal response to the ER stressor tunicamycin [54]. (iv) Others have reported similarly that antimycin A (an ETC inhibitor) caused tumor cell death and blockage of GRP78 induction under hypoglycemic conditions, but not in combination with tunicamycin or thapsigargin [54]. (v) As well, rotenone and antimycin A proved effective in the same screening assay that had been used to identify VCD and many other GRP78 downregulators: both compounds inhibited activation of a GRP78-luciferase construct under hypoglycemic conditions [54]. Altogether these results provide evidence that a functional respiratory chain is required for hypoglycemia-stimulated GRP78 expression and mounting of the ER stress response in general. This view is fully consistent with textbook knowledge [55] that simultaneous blockage of glycolysis and OXPHOS results in inevitable depletion of the cellular ATP pool, and no conveivable cellular defense mechanism is able to overcome the cytotoxic consequences when both energy sources remain blocked at the same time. Even autophagy, the cellular defense mechanism against nutrient deprivation, ceases to function when ATP levels drop too severely [51].

It thus emerges that the GRP78-luciferase-based screening assay that has been used to identify GRP78 downregulators under hypoglycemic conditions is effective at yielding compounds that block mitochondrial function. In fact, several of the GRP78 downregulators were known to inhibit mitochondrial ATP synthesis long before they were identified in this screening assay. Decades ago, efrapeptin J has been characterized as an inhibitor of OXPHOS [56], and piericidin A [57] and valinomycin A [58], [59] were shown to block ATP synthesis. Rottlerin has been characterized as a mitochondrial uncoupler [60]. Prunustatin and JBIR-type compounds are chemically very similar to the potent ETC inhibitor antimycin A [18], [58]. Arctigenin [61], pyrvinium [62] and metformin [63], [64] have demonstrated ETC inhibitory activity. Deoxyverrucosidin [17] and the central compound of our study, VCD, are members of the aurovertin family of natural compounds and share common structural features with aurovertin [65], [66] and citreoviridin [67], [68], which are highly potent inhibitors of ATPase activity. Thus, most of the purported GRP78 downregulators display verified or highly probable impairment of mitochondrial function and ATP synthesis. Conversely, classic OXPHOS inhibitors (rotenone, antimycin A) were shown to exert GRP78 downregulatory activity when tested in the GRP78-luc screening assay [54] and, as we demonstrate, rotenone faithfully mimics each and every effect of VCD that we have investigated in this present study (Figures 6, 7, 9, 10, 11).

Is it possible that inhibition of GRP78 somehow is involved in the down-regulation of mitochondrial ATP synthesis, i.e., that inhibition of OXPHOS is secondary to transcriptional blockage of GRP78 by these purported GRP78 downregulators? This is not likely for the following three reasons. (i) In the case of VCD, the time course of events is reverse. Inhibition of mitochondrial oxygen consumption (OCR) by VCD takes effect within minutes (Figure 8), and decreased ATP levels can be detected as early as 2 hours (Figure 7). In comparison, stimulation of GRP78 expression in response to hypoglycemia is a slow process, where GRP78 protein levels hardly increase within the first 8 hours (Figure 2); therefore, transcriptional blockage by VCD would not be able to affect the levels of GRP78 (a long-lived protein) at early time points where mitochondrial function already is impacted. (ii) Impairment of mitochondrial function (reduction of OCR) by VCD also occurs in the presence of plentiful glucose (Figure 8), where there is no detectable effect of VCD on GRP78. (Reduction of OCR in the presence of glucose does not lead to substantial loss of ATP, because of glycolytic compensation, as indicated by increased ECAR in Figure 8, and the literature, e.g., [58], [69]). (iii) Potent inhibition of complex I of the electron transport chain by VCD can be verified in isolated mitochondria (Figure 9), i.e., under conditions where VCD is entirely unable to impact GRP78 expression.

The repositioning of VCD–and possibly most other members of the above presented group of GRP78 downregulators–as mitochondrial inhibitors would not minimize their therapeutic potential. In fact, several of the purported GRP78 downregulators, for example, arctigenin [70], metformin [71], [72], pyrvinium [22], and versipelostatin [25], already have shown anticancer activity in vivo. As well, based on cancer’s sweet tooth [73], one would expect that preferential uptake of 2-DG by tumor cells (and resulting inhibition of glycolysis) should prime malignant cells to become exquisitely sensitive to killing by such mitochondrial inhibitors. Indeed, bona fide mitochondrial inhibitors, such as rotenone, antimycin A, or mito-carboxy proxyl (Mito-CP) have revealed promising anticancer activity in vivo when combined with 2-DG [74], [75], and this outcome has also been reported when 2-DG was combined with metformin [76]. Altogether, these results may form the basis for an anti-tumor strategy that hits both glycolysis and OXPHOS.
